# Literature review and proposal of best practice for ophthalmologists: monitoring of patients following intravitreal brolucizumab therapy

**DOI:** 10.1007/s11845-022-02929-8

**Published:** 2022-02-01

**Authors:** Dara J. Kilmartin

**Affiliations:** 1 Royal Victoria Eye & Ear Hospital, Adelaide Road, Dublin, Ireland; 2 School of Medicine, University College Dublin, Dublin, Ireland

**Keywords:** Anti-VEGF, Brolucizumab, Intraocular inflammation, Retinal occlusion, Retinal vasculitis, Safety findings

## Abstract

Brolucizumab is a novel humanised, single-chain, variable fragment inhibitor of Vascular Endothelial Growth Factor-A for the treatment of neurovascular age-related macular degeneration. Brolucizumab gained US Food and Drug Administration and European Medicines Agency approval following the Phase III HAWK (NCT02307682) and HARRIER (NCT02434328) trials which compared brolucizumab with aflibercept, presenting a tolerable safety and favourable efficacy profile. The mean change (least squares [LS] mean ± standard error) in best-corrected visual acuity letters from baseline to week 96 in the HAWK trial was 5.9 ± 0.78 for brolucizumab (6 mg) versus 5.3 ± 0.78 for aflibercept, and in the HARRIER trial, 6.1 ± 0.73 (6 mg) for brolucizumab (6 mg) and 6.6 ± 0.73 for aflibercept. Within both trials, greater reductions were noted in the central subfield thickness from baseline to week 96 in the brolucizumab (6 mg) groups versus the aflibercept group. Subsequent post-marketing reports detailed intraocular inflammation (IOI) after brolucizumab treatment and in response an independent safety review committee conducted a post hoc data review. While comparable, the rate of brolucizumab-associated IOI was higher in the post hoc analysis than the trials (4.6% and 4.4%, respectively). Findings from trials and real-world data indicate there may be pre-defining risk factors that predispose patients to IOI following brolucizumab treatment. With a thorough understanding of IOI classification and best practice management, ophthalmologists can use brolucizumab confidently and, should a case arise, they should act quickly to prevent vision loss. Herein, we provide information and guidance to support clinical decision-making related to brolucizumab use.

**Supplementary Information:**

The online version contains supplementary material available at 10.1007/s11845-022-02929-8.

## Background

Age-related macular degeneration has been found to be a leading cause of visual impairment and blindness in individuals over 40 years of age in developed countries, especially those of European descent [1–4]. In the Republic of Ireland in particular, the estimated prevalence of age-related macular degeneration within the population has been reported to be 7.2% [5]. A previous study in Ireland found that of the individuals who have previously registered as blind, age-related macular degeneration was the leading cause in those ≥ 65 years [6].

Characterised by inflammation and the presence of drusen, the disease is chronic, and generally progresses asymptomatically for numerous years. Age-related macular degeneration has been separated into distinct categories based on the likelihood of developing vision loss, and neovascular age-related macular degeneration (nAMD) presents as the most severe form, associated with retinal haemorrhage [7, 8]. While aspects of the pathogenesis are still not well understood, the presence of the protein Vascular Endothelial Growth Factor (VEGF-A), has been determined to be a pivotal factor responsible for the ocular angiogenesis seen in patients with nAMD and is a major cause of the vision loss associated with the disease [9, 10].

Brolucizumab (Beovu®, Novartis) is a novel humanised, single-chain, variable fragment inhibitor of VEGF-A and is administered via intravitreal injection [11, 12]. It binds with high affinity to all isoforms of VEGF-A, preventing the ligand-receptor interaction of VEGF receptors VEGFR1 and VEGFR2 and thus effectively suppresses the activation of the VEGF pathway [9, 13] which is responsible for the pathologic angiogenesis seen in nAMD. With a molecular weight of approximately 26 kDa, brolucizumab has been engineered with a smaller molecular mass than either aflibercept or ranibizumab, and, due to brolucizumab’s high solubility, can be delivered at a higher concentration, potentially resulting in a longer-lasting effect [9].

## Phase III clinical trial findings for brolucizumab

The HAWK (NCT02307682; *N* = 1078) and HARRIER (NCT02434328; *N* = 739) trials were pivotal, Phase III, 96-week, randomised, active-controlled, double-masked, multicentre studies that compared the efficacy and safety of brolucizumab versus aflibercept in patients with nAMD [14, 15]. The intravitreal (IVT) injections of brolucizumab (3 mg or 6 mg in HAWK; 6 mg only in HARRIER) were given initially as 3 monthly loading doses followed by an injection every 12 weeks or, if disease activity was detected, every 8 weeks. Aflibercept was administered according to the label at the time of study initiation, starting with 3 monthly injections, followed by dosing every 8 weeks [14, 15].

Brolucizumab (6 mg) was found to be an effective treatment option in nAMD, providing noninferior best-corrected visual acuity (BCVA) gains and superior anatomical outcomes when compared with aflibercept in the HAWK and HARRIER studies [14, 15]. From baseline to week 96, the mean change (least squares [LS] mean ± standard error) in BCVA letters was 5.9 ± 0.78 [6 mg] for brolucizumab compared with 5.3 ± 0.78 letters for aflibercept in the HAWK trial. In the HARRIER trial, the change in BCVA letters from baseline to week 96 for brolucizumab was 6.1 ± 0.73 [6 mg] letters and for the aflibercept group, 6.6 ± 0.73 letters [14]. Anatomical changes were noted, with greater reductions in the central subfield thickness from baseline to week 96 shown in the brolucizumab group (LS mean [6 mg] − 174.8 µm) than aflibercept (LS mean − 148.7 µm) in the HAWK trial (95% CI: 46.2 to − 5.9 µm; *P* = 0.0115) as well as the HARRIER trial (brolucizumab LS mean [6 mg] − 197.7 µm versus aflibercept − 155.1 µm; 95% CI: − 62.0 to − 23.3 µm; *P* < 0.0001). Within both trials, significantly fewer brolucizumab-treated eyes showed intraretinal or subretinal fluid at 96 weeks. The proportion of eyes with intraretinal or subretinal fluid in the brolucizumab (6 mg) group was 24% (*P* = 0.0001) compared with 37% for aflibercept. Within the HARRIER trial, the proportion of eyes showing intraretinal or subretinal fluid in the brolucizumab (6 mg) group was 24% (*P* < 0.001) compared with 39% of the aflibercept group. At week 92, it was determined that the probability of maintaining the every 12-week dosing regimen for the patients taking brolucizumab (6 mg) was 45.4% in the HAWK trial and 38.6% in the HARRIER trial.

With respect to adverse effects, the phase III trials reported the risk of intraocular inflammation (IOI) was analogous to existing anti-VEGF drugs at 2–4% [16–18]. As the proportion of eyes that lost ≥ 15 letters were comparable between the brolucizumab- (6 mg) and aflibercept- (2 mg) treated groups at week 96, these trials concluded that brolucizumab exhibited an overall well-tolerated safety profile. Based on the findings from these trials, brolucizumab was approved for use by the FDA in treating nAMD in the USA in October 2019 [19], and by the EMA in February 2020 [20].

## Post-marketing reports of intraocular inflammation

Following the release of brolucizumab onto the market, post-marketing reports were presented detailing cases of IOI. In February 2020, the American Society of Retina Specialists (ASRS) circulated a safety update which reported 14 isolated episodes of treatment-associated non-occlusive and occlusive retinal vasculitis, not previously recorded in the usual MedDRA (Medical Dictionary for Regulatory Activities) classifications for uniform coding of adverse events in clinical trials [11, 21–23]. Of these reported cases, 11 were diagnosed as occlusive retinal vasculitis that could potentially lead to vision loss. This prompted a thorough review of the available data to better understand the incidence of IOI following brolucizumab treatment. Since this time, Novartis has maintained regular updates on post-marketing case reports [http://www.brolucizumab.info] [24].

## Intraocular inflammation

Various inflammatory conditions encompass IOI including bacterial endophthalmitis, which is associated with all intraocular injections, and isolated cases of retinal vasculitis or vascular occlusion have previously been reported with anti-VEGF therapy [16, 17, 25]. The mechanism behind IOI is not fully understood. The adverse events may be autoimmune or autoinflammatory in nature and as with other biological treatments, the significance of anti-drug antibodies remains unclear [26].

## Findings from the independent safety review

Following the post-marketing reports, Novartis commissioned an independent, external safety review committee (SRC) to carry out an analysis and evaluation of the reported post-market cases [27] as well as a thorough, retrospective review of the clinical data and imaging reports from the phase III trials [21, 28]. The SRC was composed of nine global retina and uveitis specialists as well as imaging and ophthalmology experts from two separate external data monitoring committees, and, additionally, an independent observer from the ASRS.

As shown in Table 1, the reported incidence of IOI in the HAWK and HARRIER week-48 data trials was 4.4% (6 mg brolucizumab) [14, 15]. However, within the post hoc analysis conducted by the SRC, new cases were identified, which resulted in a comparable though slightly higher incidence rate of 4.6% [14, 15, 28]. Of the 50 eyes reported to have brolucizumab-related IOI, 36 eyes had concomitant retinal vasculitis (3.3%), and of these, 23 had concomitant retinal vascular occlusion (2.1%) [29]. As the focus of the post hoc analysis was on investigating the cases of IOI following brolucizumab treatment, comparisons were not made with incidence of IOI in the aflibercept group [29].

**Table 1:** Intraocular inflammation findings between the trial investigators and SRC for the HAWK and HARRIER Phase III trials (*N* = 1088)

**Brolucizumab (6 mg) *n* (%)**	
**Investigator-reported findings**	
Cases of IOI	(4.4)
**SRC findings**	
Cases of IOI	50 (4.6)
Concomitant retinal vasculitis	36 (3.3)
Concomitant retinal vasculitis and retinal vascular occlusion	23 (2.1)

Abbreviations: *IOI*, intraocular inflammation; *SRC*, safety review committee

Despite the risk of vision loss associated with these events following brolucizumab injection, the overall rate of at least moderate vision (≥ 15 ETDRS letters) loss in the original HAWK and HARRIER trial population assessment was comparable between the brolucizumab (7.4%) and aflibercept (7.7%) treatment arms [14, 28]. As shown in Table 2, the incidence of at least moderate loss of visual acuity (≥ 15 letters) as a result of IOI associated with brolucizumab, during the observation period was 0.74% (8 of 1088 patients) [28].

**Table 2:** Cases of IOI with visual acuity loss in the HAWK and HARRIER trials (adapted from [29]; *N* = 1088)

**Event in cases with IOI**^**a**^	**Incidence *n* (%)**^**b**^
Visual acuity loss	
At least moderate	8 (0.74)
Severe	5 (0.46)
Visual acuity loss with retinal vasculitis	
At least moderate	8 (0.74)
Severe	5 (0.46)
Visual acuity loss with retinal vasculitis and retinal occlusion	
At least moderate	7 (0.64)
Severe	5 (0.46)

^a^At least moderate visual acuity loss defined as ≥ 15 EDTRS letter loss; severe visual acuity loss defined as ≥ 30 EDTRS letters

^b^Numbers reported are those which were categorised as “definitely drug related; within the spectrum” or “probably drug related; probably in the spectrum”

Abbreviations: *IOI*, intraocular inflammation

The SRC also observed that almost half of brolucizumab-related inflammatory events (48%) were initially diagnosed in the first 3 months of therapy and almost three-quarters of the cases were diagnosed within 6 months of therapy (74%) [28]. A further 12% presented between 12 and 18 months after treatment. Given the delay in the occurrence of IOI following treatment with brolucizumab, it is unlikely that the cases reported were a direct result of a reaction to the injection itself or drug toxicity. Interestingly, patients whose first IOI-related event occurred more than 12 months after the first injection had no reported vision loss at the end of the study [28]. Furthermore, the SRC found that all patients who experienced retinal artery occlusion demonstrated cardiovascular comorbidities such as hypertension or cardiac arrhythmias [28].

Based on the SRC’s review of post-marketing cases, Novartis initiated an update to the Summary of Medical Product Characteristics (SmPC) [20, 28]. This label safety update, i.e. “retinal vasculitis and/or retinal vascular occlusion, typically in the presence of IOI”, has been approved by several health authorities, including the FDA and EMA and is also included in the SmPC [20].

## Real-world data on IOI following brolucizumab treatment

As there are limited clinical trial data, real-world data on IOI following brolucizumab treatment offer valuable information which may further inform ophthalmologists. At the American Academy of Ophthalmology (AAO) 2020 Annual Meeting, baseline patient characteristics were presented which were found to be potentially associated with the incidence of inflammation-related adverse events which may occur following treatment with brolucizumab. In the analysis of US Real-World and Phase III data which included 12,000 patients from the Intelligent Research in Sight (IRIS) registry, it was found that those with the highest observed risk for experiencing retinal vasculitis and/or retinal vascular occlusion in the 6 months after the first treatment with brolucizumab had prior IOI and/or prior retinal vascular occlusion in the 12 months before first brolucizumab injection [30]. Against an observed overall retinal vasculitis/retinal vascular occlusion risk rate of 0.46% for all brolucizumab-treated patients in the registry, individuals with prior IOI and/or retinal vascular occlusion had an increased risk of 3.97% [30].

In the retrospective study of IOI cases following brolucizumab treatment collected by the ASRS, the frequency of symptoms presented within post-marketing cases were reported as 62% blurred vision, 46% floaters, 31% pain, 19% redness, and 12% scotoma. It was also found that the location of the IOI varied. Within 8 eyes it was anterior (31%), posterior in 7 eyes (27%), and both anterior and posterior in 9 eyes (35%) [23]. Posterior vasculitis includes retinal vasculitis, which can occur with or without occlusion (especially of the arterial vessels) [23]. It was observed that non-occlusive or occlusive vasculitis was typically preceded by non-specific signs of IOI (e.g. keratic precipitates and intraocular cells or flares) [23]. Importantly, the majority of brolucizumab-treated patients with IOI did not develop vasculitis during the observation period [23].

The SRC found that anterior or intermediate uveitis did not affect visual acuity in the cases presented [28]. In patients with non-occlusive and occlusive vasculitis, visual acuity was not always reduced [23, 28]. A reduction in visual acuity occurred where the vasculitis involved branches of the retinal vessels supplying the optic nerve head or macula. In isolated cases, this led to a severe reduction in visual acuity [23, 28]. The mean (range) number of days from the last injection to the onset of IOI in these events was 25.5 (1–91). For those patients with both IOI and retinal vasculitis, the mean number of days was 22 (1–49) and for those with IOI as well as retinal vasculitis and retinal occlusion, the mean number of days was 25 (3–49) [28].

Adding to the body of information are case-study series largely reporting single-centre experience. A report of 3 eyes from 3 Japanese patients indicated that patients noted symptoms of blurry vision and floaters 11–18 days after their first injection with brolucizumab (two of the patients had previously been treated with aflibercept) [31]. After investigation, retinal vasculitis was diagnosed and treated with systemic steroid therapy, with visual acuity restored in all eyes 6 weeks after symptom onset. A case series of 15 eyes from 12 female, treatment-experienced patients reported that retinal vasculitis and IOI were diagnosed at a mean of 30 days post-brolucizumab injection [32]. The patients reported floaters (*n* = 8), reduced or blurred vision (*n* = 7), scotoma (*n* = 3), or ocular discomfort (*n* = 2), and all eyes showed vitreous cells or opacities, with anterior chamber cells reported in 11 eyes (73.3%). Treatment primarily consisted of corticosteroids (systemic, intravitreal, and topical). After a mean follow-up of 25 days, mean visual acuity was 20/136, a significant decrease compared to baseline (20/53; P = 0.033). A retrospective analysis of 127 eyes from 127 consecutively treated patients in Japan included 43 patients (33.9%) who were treatment naive and 84 (66.1%) were switch patients [33]. From this cohort, 12 patients (9.4%) experienced an IOI, 4 eyes (3.1%) developed retinal vasculitis, and 2 eyes (1.6%) developed retinal vasculitis and vascular occlusion. IOI developed in 9 eyes (75%) after a mean of 23.2 ± 9.3 days following the first brolucizumab injection (range 10–36 days). The remaining 3 (25%) occurred after second or third injections. IOI in all eyes was treated with topical corticosteroids (0.1% betamethasone). Retinal vasculitis cases were additionally treated with sub-tenon’s injection of triamcinolone acetonide (20 mg). Inflammation resolved within 2 months in all patients. Such reports will continue to contribute to the growing knowledge on incidence and management of IOI following treatment with brolucizumab.

### Patient selection

**Fig. 1:**
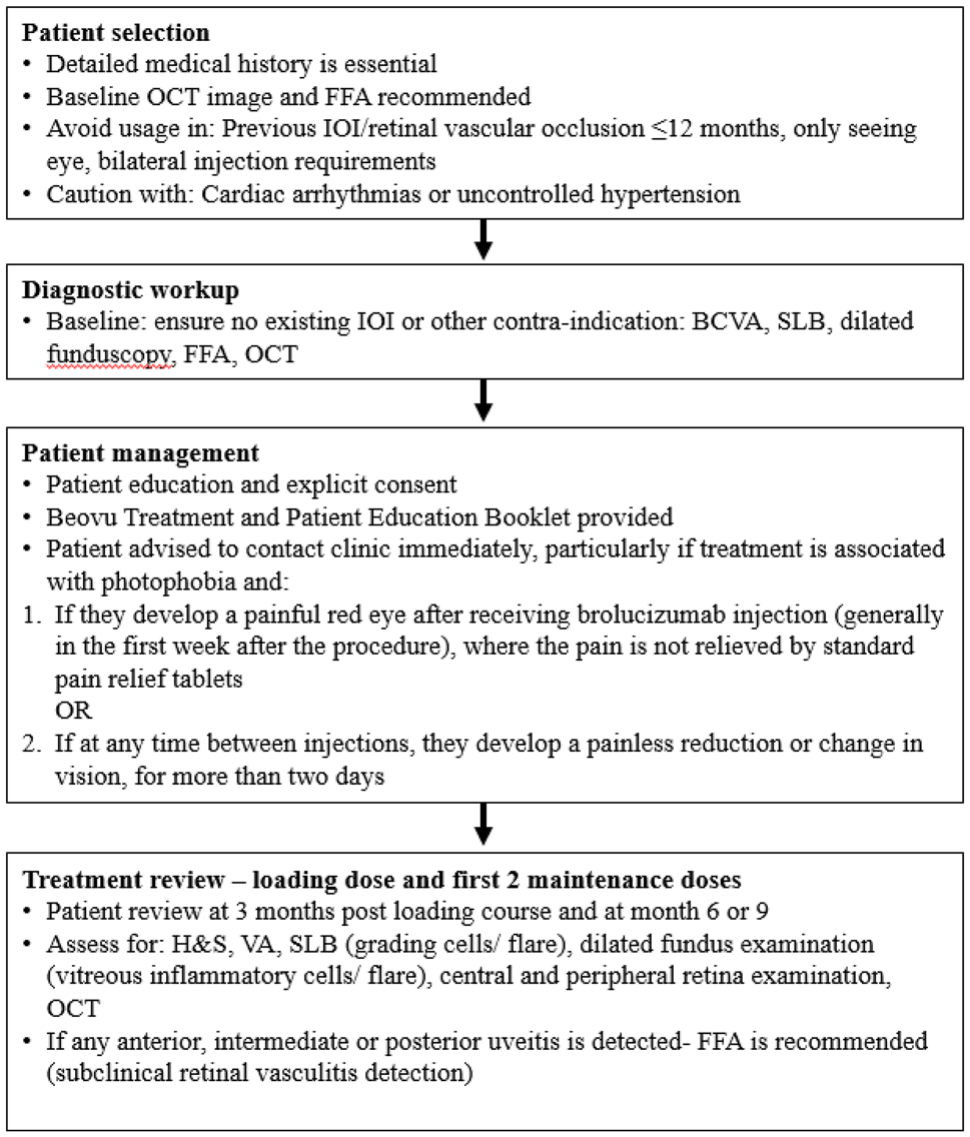
Brolucizumab treatment pathway. Abbreviations: BCVA, best-corrected visual acuity, H&S, history and symptoms, FFA, fundus fluorescein angiography, IOI, intraocular inflammation OCT, optical coherence tomography, SLB, slit lamp biomicroscopy, VA, visual acuity

Based on the available data, risk factors have been identified which may inform clinical decision making. As per Fig. 1, before giving an anti-VEGF injection for the treatment of nAMD, the ophthalmologist should ensure that no existing active IOI or other contraindications are present [21]. Patients who may not be suitable for treatment with brolucizumab include those with previous uveitis (especially in the last 12 months), those with bilateral wet AMD, and patients with uncontrolled hypertension or cardiac arrhythmias [28, 34].

The longer treatment interval provided by brolucizumab compared with other treatments may make it the treatment of choice for certain patients [35, 36]. Ideally, patients should have the following baseline tests prior to beginning brolucizumab treatment [21]:

- BCVA (ETDRS chart)
- Slit lamp biomicroscopy (anterior/intermediate segment examination and grading of anterior chamber cells, flare or keratic precipitates
- Dilated retinal examination/colour fundus photography
- Fundus fluorescein angiography (peripheral sweeps)
- Optical coherence tomography (OCT)

### Patient management—education and explicit consent

Comprehensive patient education is essential for early detection of IOI. In addition to routine education on the symptoms of post-IVT bacterial endophthalmitis, prior to commencing therapy, patients should be made aware of the risks of developing IOI following brolucizumab injections and should have provided explicit consent for treatment [21, 26].

Where bacterial endophthalmitis may be present at approximately 3–7 days post treatment, non-infectious IOI can develop over a longer period [21, 26, 28]. Consequently, relevant symptoms or signs of inflammation within 7 days of IVT with brolucizumab are more likely to be indicative of bacterial endophthalmitis, than non-infectious IOI. Initially, symptoms for both adverse events may be similar, which can make differentiating between the conditions difficult [21, 26]. It is recommended that any patient experiencing floaters or ocular discomfort, persisting for more than 2 days, should be investigated for the presence of IOI [26].

Non-infectious uveitis is characterised by a reduction in visual acuity caused by the infiltration of inflammatory cells (myopic or hyperopic shift). Other symptoms include photopsia, scotomas, and vitreous opacities [35, 36]. Symptoms of intermediate or posterior uveitis depend on the site of inflammation. It is not uncommon for peripheral lesions to be asymptomatic, with none of the typical symptoms or clinical findings (including redness and pain) observed in acute anterior chamber inflammation [26, 35, 36]. Patients should be made aware of the need to contact their ophthalmologist immediately, even if only minor changes or symptoms are experienced [21, 26]. A patient questionnaire may be beneficial in elucidating relevant ocular symptoms of concerned patients **(**Online resource 1) [34]. It is recommended that ophthalmologists weigh the benefits of using brolucizumab and the risk of developing IOI in their patients and act according to their best judgement [27].

### Treatment review

As non-infectious IOI associated with brolucizumab occurs over a longer period of time than infectious IOI, modifications to the current IVT follow-up regime are not necessary, even for asymptomatic patients [26]. However, ophthalmologists involved in follow-up care should be aware of the need for precautionary IOI monitoring measures [26]. As per Fig. 1, examinations should be performed at baseline, 3 months post loading course, and at month 6 or 9 (at the end of two further 8 or 12 weekly injection intervals). The timings chosen are based on the findings from the SRC [28] and should allow for the early detection of possible uveitis/vasculitis without overburdening the patient or ophthalmologist. Each examination should include:

- Careful, magnified, oblique high-intensity beam slit lamp assessment of the anterior chamber (looking for inflammatory cells and flare)
- High magnification dilated fundoscopy assessment of the:
  - Anterior and posterior vitreous (vitreous inflammatory cells and haze)
  - Central and peripheral retina (retinal haemorrhages, cotton wool spots, dilated retinal veins, optic disc and macular oedema, choroiditis)
  - Pars plana (snow banking) assessment

### Diagnosing and managing uveitis and vasculitis

In the event that a patient being treated with brolucizumab does present with a suspected inflammatory condition, the ophthalmologist should be familiar with the objectives of effective IOI management: to demonstrate the presence of inflammatory activity, determine the location and severity, preserve or restore visual acuity, and prevent or ensure the earliest possible treatment for complications [9].

As shown in Table 3, signs and symptoms as well as the primary sites of inflammation may differ depending on the specific inflammatory condition. If at any point a patient develops features associated with IOI, they should be classified and treated according to the available guidelines [26, 35–37]. As with posterior uveitis, panuveitis may present with vasculitis and/or vascular occlusion with possible retinal and choroidal involvement. Unlike posterior uveitis, however, inflammation may be detected in the anterior chamber vitreous body and retina or choroid.

**Table 3:** Anatomical uveitis classification and distinguishing features of uveitis and vasculitis [26, 35–37]

**Where intermediate uveitis patient is developing features of posterior uveitis, refer to uveitis specialist**			
	**Anterior uveitis**	**Intermediate uveitis**	**Posterior uveitis/non-occlusive/occlusive vasculitis**
**Signs and symptoms**	• Floaters • Sudden reduced vision • Distortion (metamorphopsia) • Pain • Photophobia • Reduced vision	• Floaters • Flashes • Reduced vision distortion (metamorphopsia)	• Floaters • Cotton wool spots • Intraretinal haemorrhages • Sudden reduced vision • Visual field defect (scotoma) • Distortion (metamorphopsia)
**Primary site of inflammation**	• Anterior chamber	• Vitreous body • Pars plana	• Retina or choroid
**Distinguishing features**	• Confined to the anterior chamber	• Vitreous white inflammatory cells, vitreal haze, snow banking with pars planitis	• Vasculitis and/or vascular occlusion with possible retinal and choroidal involvement
**Recommended examination technique**	• VA, SLB (grading of cells/flare in AC) • Dilated fundoscopy (vitreous body/retinal periphery) • Slit lamp biomicroscopy • Intraocular pressure • Binocular examination of vitreous body	• VA, IOP, OCT, SLB (anterior and intermediate) • Dilated fundoscopy (vitreous body/central retina and periphery) • CFP of retinal periphery • FFA with peripheral sweeps • Fluorescein angiography	• VA, IOP, SLB, dilated fundoscopy • Requires differentiation: arterial V venous, central V peripheral • FFA with peripheral sweeps • Widefield OCT and CFP retina (and periphery)
**Findings**	• Inflammatory cells/flare in AC and possible spillover into vitreous (monitor) • KPs • Iritis • Iridocyclitis • Anterior cyclitis	• Inflammatory cells in vitreous body/pars plana—possible vitritis • Grade vitreous opacification (SLB) • Possible vascular sheathing/macular oedema • If drug-associated inflammation suspected—FFA with peripheral sweeps • Pars planitis • Posterior cyclitis • Hyalitis	• Arterial—CWS, white retinal infiltrates • Retinal whitening •Retinal venous dilation and segmentation • Cherry Red Spot • Vascular non-perfusion • ONH and macular oedema • If venous—intraretinal haemorrhages, irregular venous calibre, arterial occlusion, ischaemic vasculitis • Focal, multifocal or diffuse choroiditis • Retinochoroiditis retinitis • Neuroretinitis • Perivunular sheathing is a sign of past inactive retinal vasculitis

Abbreviations: *AC*, anterior chamber; *CAI*, carbonic anhydrase drop; *CFP*, colour fundus photograph; *C/I*, contraindicated; *CWS*, cotton wool spot; *FFA*, fundus fluorescein angiography; *IOP*, intraocular pressure, *IVT*, intravitreal; *KP*, keratic precipitate; *OCT*, optical coherence tomography; *ONH*, optic nerve head; *PRP*, pan-retinal photocoagulation; *SLB*, slit lamp biomicroscopy

## Clinical findings and recommended procedure

### Anterior uveitis

In the event that an ophthalmologist suspects anterior uveitis, a slit lamp examination should be used to isolate keratic precipitates and/or cells in the anterior chamber. Additionally, in these patients, a dilated fundus examination is required to evaluate the vitreous body and retina, including the periphery. This is essential in conclusively ruling out vasculitis [26]. Fluorescein angiography may be used to detect possible subclinical retinal vasculitis which is potentially the most sight-threatening form of IOI. In anterior uveitis, isolated inflammatory cells may be present in the anterior vitreous segment, because of spill over. These should not increase in number in subsequent examinations, but should they do so, further investigations (including the use of fundus fluorescein angiography) are necessary [26].

### Intermediate uveitis

The primary site of inflammation in intermediate uveitis is the vitreous body and/or pars plana, although peripheral venous vascular sheathing and macular oedema may also be involved in some cases [25]. A semi-quantitative grading of the degree of inflammation (anterior chamber cells, flare) with fluorescein angiography is recommended to monitor the progression of any inflammation which arises. In some cases, other inflammatory features can help in making a differential diagnosis [26]. If drug-associated IOI is suspected, FFA (with peripheral sweeps) is indicated. It is essential that the retinal periphery is imaged. Intermediate uveitis can only be assessed properly with a dilated pupil examination [21, 26].

### Posterior uveitis

For the diagnosis of posterior uveitis, the FFA provides key information for assessing retinal and choroidal involvement as well as structural complications. This information can also be relevant during follow-up and can assist with the identification of any inflammatory changes in the retina, choroid, optic nerve head, and retinal vasculature, as well as the identification of any typical complications (such as macular oedema, papilloedema, and vascular occlusion). In addition, the FFA may provide information on inflammatory activity (including inflammatory lesions such as vascular leaks, alterations of retinal pigment epithelium or macular oedema) and may be used to diagnose macular oedema which may not be detectable with the use of OCT [21, 26].

### Retinal vasculitis

Early diagnosis and intervention are particularly important for retinal vasculitis, as this condition is potentially sight threatening. Unlike other manifestations of IOI, retinal vasculitis is not included in the SUN classification [26]. To date, the few cases of retinal vasculitis observed after administration of brolucizumab have typically taken the form of peripheral, segmental vasculitis characterised by the presence of segmental vascular sheathing [21, 26]. The spectrum ranges from peripheral vascular involvement to arterial occlusion. If arterial occlusion affects the vascular supply to the optic nerve or macula, severe loss of function, with loss of visual acuity and visual field defects, may occur. Brolucizumab-associated ischaemic vasculitis may also be reported as cotton wool spots and intraretinal haemorrhages [21, 26]. To ensure best visual outcomes for all treated patients, where intermediate uveitis appears to be progressing towards vasculitis, the patient should ideally be immediately referred to a uveitis specialist [21, 26].

## Treatment for IOI following brolucizumab treatment

Treatment for IOI varies depending on the site of inflammation and while treatment guidelines are available (Table 4), there is a lack of real-world evidence which indicates optimal treatment durations with any certainty for IOI following brolucizumab treatment [26].

**Table 4:** Treatments based on anatomical uveitis classification

**Anterior uveitis**	• Topical corticosteroid drops: 1% prednisolone acetate, 0.1% dexamethasone • Hourly dosing and reduce on improvement (generally treat for several weeks) • Possibly also mydriatic drop (ciliary spasm) • Monitor IOP (may require BB or CAI drop) and taper dose before stopping • Weaker active substance in steroid responders
**Intermediate uveitis**	• If macular oedema/pronounced vitritis: treat intermediate uveitis with systemic corticosteroids • In acute stage: oral corticosteroid, with either: • Prednisolone equivalent, 1 mg/kg body weight • IV corticosteroids (where necessary): methylprednisilone 10–30 mg/kg body weight, for 3 days (max daily dose is 1 g), for approximately 6–12 weeks; taper to a maintenance dose of 0.1 mg/kg body weight if required (taper for withdrawal). Higher dose/longer taper may be justified • Immune-modulating agents (DMARDS) in certain cases • Where corticosteroid therapy is C/I, steroid IVT • PRP for retinal neovascularisation or significant peripheral retinal capillary non-perfusion/ischaemia not advised unless non-clearing vitreous haemorrhage • Vitrectomy not advised unless vitreous haemorrhage
**Posterior uveitis/non-occlusive/occlusive vasculitis**	• In acute stage: oral corticosteroid, either: • Prednisolone equivalent, 1 mg/kg body weight • IV corticosteroids (methylprednisilone 10–30 mg/kg body weight, for 3 days (max daily dose is 1 g), for approx. 6–12 weeks; taper to a maintenance dose of 0.1 mg/kg body weight if required (taper for withdrawal). Higher dose/longer taper may be justified • Immune-modulating agents (DMARDS) in certain cases • Where corticosteroid therapy is C/I, steroid IVT • PRP for retinal neovascularisation or significant peripheral retinal capillary non-perfusion/ischaemia not advised unless non-clearing vitreous haemorrhage • Vitrectomy not advised unless vitreous haemorrhage

Abbreviations: *BB*, beta blocker eye drop; *CAI*, carbonic anhydrase drop; *C/I*, contraindicated; *DMARDS, IOP*, intraocular pressure; *IV*, intravenous; *IVT*, intravitreal; *PRP*, pan-retinal photocoagulation

### Anterior uveitis

For anterior uveitis, the inflammation limited to the anterior chamber can usually be managed with topical therapy and should always be treated. Guidelines on treating an acute episode of anterior uveitis recommend corticosteroids, preferably with a highly potent active substance. It is recommended that initially, treatment be given frequently (e.g. hourly) and should generally be continued for several weeks. Dosing may be adapted depending on a patient’s progress. To rule out steroid or inflammation-induced ocular hypertension, it is recommended that ophthalmologists monitor intraocular pressure and use medication to reduce intraocular pressure if required [21, 26, 35].

### Intermediate uveitis, posterior uveitis, and retinal vasculitis

Intermediate uveitis should always be treated where there is macular oedema or pronounced vitritis. In cases of intermediate uveitis with vasculitis, posterior uveitis, or isolated retinal vasculitis following IVT with brolucizumab, the primary treatment should be systemic corticosteroids [21, 26, 35, 36]. The treatment of posterior uveitis or retinal vasculitis should be aligned to the recommendations given in the guideline on intermediate uveitis [21, 26, 35, 36]. In the acute stage of intermediate uveitis, oral (initially approx. 1 mg/kg body weight prednisolone equivalent) or where necessary intravenous corticosteroids are recommended. On an individual basis, higher doses and a longer time to taper may be justified. While there is presently insufficient experience with immune-modulating drugs (DMARDs), ophthalmologists may choose to use them on a case-by-case basis [26]. In situations in which a systemic corticosteroid therapy is contraindicated, IVT with an off-label steroid may be considered. Pan-retinal photocoagulation is recommended in patients with secondary retinal neovascularisation; however, it should be noted that vitrectomy is not recommended without the presence of vitreous haemorrhage [21, 26].

## Conclusion

nAMD is a considerable cause of vision loss, especially for those of European descent, and brolucizumab has been shown to offer significant advantages over other treatments, with many patients in the trials achieving longer intervals between injections as well as noninferior visual gains and superior anatomical outcomes compared with aflibercept. Due to the risk of bacterial endophthalmitis associated with anti-VEGF IVT, patients and ophthalmologists should be well versed in the symptoms of IOI. The cases of IOI necessitate that ophthalmologists carefully weigh the potential risks as well as the benefits of using brolucizumab. Patients should be provided with appropriate information on the signs and symptoms of IOI, and their explicit consent must be given prior to treatment. If IOI is detected post treatment, the detailed guidelines provided can be applied to differentiate the distinct conditions and guide case management [10, 11]. Brolucizumab is a promising treatment against a potentially debilitating condition and ophthalmologists should act according to their best judgement.

## Supplementary Information

Below is the link to the electronic supplementary material.

Supplementary file1 (DOCX 14 KB)

## Data Availability

NA.

## Code availability

NA.
