# Supplementary material for: Literature review and proposal of best practice for ophthalmologists: monitoring of patients following intravitreal brolucizumab therapy
Source: Ir J Med Sci. 2022 Feb 1;192(1):447–56. doi: 10.1007/s11845-022-02929-8 (PMC9892069; doi:10.1007/s11845-022-02929-8)
Supplement: Supplementary file 1 — Supplementary file1 (DOCX 14 KB) [file 11845_2022_2929_MOESM1_ESM.docx]

**Supplementary material**

## Suggested content for a patient questionnaire:

Since your last eye injection, in the treated eye:

- Has your vision become more blurred?
- Have you noticed black flecks (floaters) in your vision?
- Has the treated eye been painful?
- Has the treated eye been more sensitive to bright light?
- Have you noticed more redness of the white of the eye?
- Have you noticed more of an increase in the patch of vision loss?
